# Analysis of SalHV-1 Genes by Structure Prediction and Comparison Shows an Expanded Core Gene Set of the Order *Herpesvirales*

**DOI:** 10.3390/v18030372

**Published:** 2026-03-17

**Authors:** Richard J. Roller, Joan Martí-Carreras, Piet Maes

**Affiliations:** 1 Department of Microbiology and Immunology, Carver College of Medicine, University of Iowa, Iowa City, IA 52242, USA; 2 Zoonotic Infectious Diseases Unit, Department of Microbiology and Immunology, Rega Institute, KU Leuven, Herestraat 49, Box 1040, 3000 Leuven, Belgiumpiet.maes@kuleuven.be (P.M.)

**Keywords:** herpesviruses, virus evolution, structural modeling and similarity search, alloherpesviruses

## Abstract

The order *Herpesvirales* contains three families, *Orthoherpesviridae*, *Alloherpesviridae*, and *Malacoherpesviridae*. The time since divergence of families from the common ancestor makes protein primary sequence comparison an insensitive tool for identifying common genes. Comparison of three-dimensional protein structures can reveal similarities that are not evident in primary sequences. Salmonid herpesvirus 1 (SalHV-1) is an alloherpesvirus. Complete sequencing of SalHV-1 VR-868 strain Winthrop by a combination of short- and long-read methods revealed 120 putative open reading frames (ORFs). BLAST search for similar protein sequences discovered five ORFs that encoded proteins with homologs in the orthoherpesviruses, including the major capsid protein, capsid triplex subunit 2, the catalytic subunit of the DNA polymerase, the helicase subunit of the helicase/primase complex, and the terminase ATPase subunit. An annotation of the ORFs of SalHV-1 was performed in which ORFs of SalHV-1 were modeled using AlphaFold3, and the models were used as prompts for structural similarity search using DALI and FoldSeek. Completion of this search strategy for the entire genome expanded the set of genes shared among the *Herpesvirales* to include additional proteins related to DNA replication and genome integrity, capsid assembly and genome packaging, and capsid nuclear egress. No homologs for any tegument proteins or proteins of the conserved entry apparatus of the *Herpesviridae* (gB, gH or gL) were discovered.

## 1. Introduction

Members of the order *Herpesvirales* are widespread in the animal kingdom, with species isolated from almost all classes of the vertebrates and from molluscan invertebrates [1]. The order is divided into three families based on sequence similarity and host range. The most familiar and best-characterized members of the order are in the family *Orthoherpesviridae* that infect mammals, birds, and reptiles. This family includes all the human herpesviruses. Members of the family *Malacoherpesviridae* infect mollusks, and those of the family *Alloherpesviridae* infect fish and amphibians. The order of divergence of the three families from each other is unclear, with some trees suggesting divergence of the alloherpesviruses from the common ancestor of the malaco- and orthoherpesviruses prior to their split, while others suggest divergence of the malacoherpesviruses from the common ancestor of the allo- and orthoherpesviruses [2,3,4,5].

While the ortho- and alloherpesviruses share a common virion morphology and similar pathways of virion morphogenesis, the molecular bases for these similarities are unclear since they share relatively few genes that are homologous based on sequence identity [6,7]. Homologous genes that have been recognized include components of the DNA replication, capsid assembly, and DNA packaging machinery [8]. Even within these categories, however, essential conserved genes of the orthoherpesviruses have not been recognized in the allo- and malacoherpesviruses, raising the possibility that they have evolved machinery that differs to a greater or lesser extent to accomplish the same goals. For example, no genes homologous to those that encode nuclear egress complex components, integral membrane or tegument proteins of the orthoherpesviruses have been observed by sequence similarity in the alloherpesviruses.

The *Alloherpesviridae* are currently divided into four genera based on host specificity: the ictaviruses that infect catfish and sturgeon species, the salmoviruses that infect salmon and trout species, the cyviruses that infect carp, and the batraviruses that infect frogs and toads (https://ictv.global/report/chapter/alloherpesviridae/alloherpesviridae, accessed on 12 February 2026). The taxonomic division based on host species is supported by differences in genome organization and by alignment analysis of twelve genes conserved among the alloherpesviruses [9]. Only seven of those twelve common genes identified are also shared with the orthoherpesviruses.

Salmonid herpesvirus 1 (SalHV-1) and the closely related SalHV-2 and -3 are members of the genus Salmovirus that are known to infect several species of salmon (*Salmo* sp.) and trout (*Oncorhynchus* sp.). While SalHV-1 is only linked to mild disease, SalHV-2 and SalHV-3 are correlated to heavy losses for wild fish populations as well as in aquaculture [6,10]. SalHV-1 spreads via ovarian and seminal fluids through natural outbreaks, which are infrequent. The virus replicates in *O. mykiss* RTG-2 cells and forms complex syncytia [11]. SalHV-1 is a relatively understudied alloherpesvirus in terms of genomics, evolutionary history, and pathogenicity. However, the ability to grow the virus in culture makes it a useful model system for understanding its more pathogenic relatives.

The failure to find many of the so-called “core genes” of the family *Orthoherpesviridae* in the allo- and malacoherpesviruses might simply be due to the ancient divergence between the families that makes it difficult to identify homologous genes by primary sequence comparison. Structural comparison is a more sensitive detector of homology than primary sequence similarity. The advent of highly accurate structural modeling by AlphaFold and its upgrades enables similarity search and discovery of distantly homologous genes without experimental structure determination [12]. Here, we have undertaken a comprehensive structure modeling-based annotation of the genome of Salmonid herpesvirus 1 (SalHV-1), an alloherpesvirus. We report both an expanded set of genes that are shared between the allo- and orthoherpesviruses (Table 1) and provide additional support for surprising absences, including all tegument and envelope proteins and some essential components of the DNA replication and DNA packaging mechanisms. These absences suggest that the alloherpesviruses may accomplish common herpesvirus processes in replication and virion morphogenesis with substantially simplified machinery. Additionally, we find interesting structural differences in the machinery of capsid assembly, DNA packaging, and nuclear egress that likely correspond to mechanistic differences.

## 2. Materials and Methods

**Virus collection and DNA extraction.** The SalHV-1 strain Winthrop was obtained from ATCC (ATCC collection VR-868, ATCC, Manassas, VA, USA). The virus was not subcultured, but DNA was directly extracted using the QIAGEN DNA Blood kit (QIAGEN, Hilden, Germany) using manufacturer’s recommendations.

**Hybrid sequencing and assembly.** Nanopore sequencing library LSK109 was prepared (Oxford Nanopore Technologies Ltd., Oxford, UK), multiplexed (EXP-NBD104) and loaded on a R9.4.1 flowcell and basecalled on a GridION, following manufacturer’s recommendations. Illumina paired end (2 × 150 bp) data was obtained using a NexteraXT DNA Sample Preparation Kit and Index Kit (Illumina Inc, San Diego, CA, USA). PhiX Control v3 was added at 5% in the sequencing library and loaded in an MiSeq v2. Sequencing data from both platforms was quality checked, assembled, and corrected using VANIR v0.2 [14]. The SalHV-1 complete genome is available on GenBank (OK337613) together with sequencing raw data (SRR16095036-SRR16095036), under BioProject PRJNA766810.

**Protein and repeat annotation.** Terminal and interspersed repeats were identified through BLAST v2.6.0 (blastn-evalue 0.001) [15]. Tandem repeats were identified with mTR (repeats > 10 bp and with >80% similarity) [16]. Open-reading frames (ORFs) were predicted GeneMarkS v4.32 [17] with a threshold ≥ 70 amino acids. An initial annotation based on sequence similarity was performed using BLAST v2.6.0 (blastp-evalue 0.001) [15].

**Modeling and DALI search and structure alignment generation**—SalHV-1 ORFs were modeled using the AlphaFold3 interface [18]. The five models produced from each prediction, and the predicted aligned error (PAE) plot were used to evaluate potential domain sub-structures within the models. In each case, the entire 0 model was used as a prompt for a heuristic PDB search using DALI [12]. In specific cases, subdomains were used as prompts for the same search. Alignments between DALI matches and SalHV-1 models were generated using the pairwise alignment tool at the Research Collaboratory for Structural Bioinformatics Protein Data Bank (RCSB PDB) site (https://www.rcsb.org/alignment, accessed 9 March 2026) using the TM-align alignment method [19].

**Determination of distribution of SalHV-1 genes across the *alloherpesviridae***—Homologs of herpesvirus genes found in SalHV-1 were used as queries for a PSI-BLAST search of the non-redundant (nr) sequence database limited to the virus taxon (taxid:10239). Matches with e-values ≥ 0.005 were not used for subsequent iterations of the search. If putative homologs were identified in all sequenced alloherpesvirus genomes, examples from each of the alloherpesvirus genera (Ranid herpesvirus 2 for the batraviruses, cyprinid herpesvirus 2 for the cyviruses and ictalurid herpesvirus 1, from the ictaviruses) were modeled using AlphaFold3 and the structures were aligned with the SalHV-1 protein to confirm the relatedness suggested by sequence alignment.

## 3. Results

### 3.1. Genome Structure

**Figure 1:**
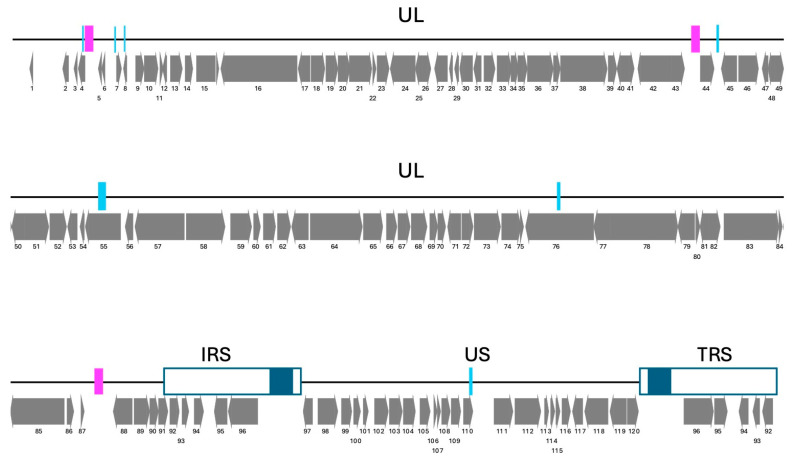
Schematic diagram of the SalHV-1 genome showing organization of long and short genome components, the position of interspersed repeats (magenta blocks), tandem repeats of varying sequences (cyan blocks), and inverted repeats that flank the S component (open boxes) that contain long non-coding sequences (dark blue boxes). Positions and orientations of ORFs are depicted as arrows below the genome schematic, with each ORF numbered from left to right.

The complete genome of the SalHV-1 was sequenced from the ATCC VR-868 strain Winthrop (BioSample SAMN2186916), which corresponds to the first-ever detected case of SalHV-1 [11]. The Winthrop strain was subsequently passaged six times in RTG-2 (Rainbow trout gonad) ATCC CCL-55 cells, before its deposition to ATCC. A mixture of SalHV-1 virions and SalHV-1 infected RTG-2 cells were obtained from ATCC for dual platform sequencing. Nanopore and Illumina sequencing yielded 1060.60× and 817.46× coverage, respectively. The reconstructed complete genome is 173,467 bp long, consistent with a previous genome size estimate of 174.4 kb based on restriction enzyme fragment analysis [20]. Repeat analysis found two long inverted repeats: the internal repeat sequence (IRS) and the terminal repeat sequence (TRS), at the right terminus of SalHV-1 of 10.6 kb, flanking a unique short (US) coding region of 25.3 kb (Figure 1). No repeated sequences were found flanking the unique long (UL) region of 126.7 kb, suggesting that SalHV-1 has a class D herpesvirus genome, in agreement with its initial characterization [20]. Structural variant analysis performed with VANIR confirmed the presence of SalHV-1 genome isomers, in which the US appears to be inverted, as expected from a class D herpesvirus genome [21]. The repeat analysis showed three interspersed repeats (IR) located in the UL region adjacent to the IRS, in the middle of the UL, and close to the UL terminus (Figure 1 magenta bars). Structural variant analysis did not show any inversions between any of the three IRs. Repeat analysis also reported nine different direct tandem repeats distributed along the genome (Figure 1 cyan bars) that might undergo expansion or contraction.

Open reading frame (ORF) prediction with GeneMarkS v4.32 [17] with a threshold ≥ 70 amino acids found 120 unique ORFs of which five (ORFs 92–96) were duplicated because they are contained within the inverted repeats that flank the US region. Annotation based on BLAST v2.6.0 searches using an E-value cutoff of 0.001 found significant matches to six proteins of the orthoherpesviruses in seven ORFs, including ORF23 (triplex subunit 2), ORF24 (maturational protease), ORF38 (MCP), ORF58 (DNA pol catalytic subunit), ORF60 (dUTPase), and ORFs72 and 82 (ATPase subunit of terminase). However, BLAST search did not find homologs to the following genes of the orthoherpesviruses: (i) any conserved component of the entry apparatus; (ii) additional components of the DNA replication apparatus, including, the single-stranded DNA binding protein, processivity factor, and any component of the heterotrimeric helicase/primase complex; (iii) conserved capsid proteins, including the scaffold, portal, and triplex 1 subunit; (iv) components of the DNA packaging apparatus, including the other two subunit of the heterotrimeric terminase complex, alkaline exonuclease, and pentameric packaging factor; (v) either component of the heterodimeric nuclear egress complex (NEC); (vi) any component of the viral tegument; (vii) any of the conserved gene expression regulators. It seemed likely that other homologs of orthoherpesvirus proteins were present, but unrecognized due to low primary sequence similarity. To address this, we used AlphaFold 3 to predict protein structure models of all SalHV-1 predicted ORFs, then used structural alignment tools to identify SalHV-1 genes that are likely homologs of core proteins in the orthoherpesviruses. We expand the list of likely core genes in the order by ten (Table 1), grouped below by viral replication stage.

### 3.2. DNA Replication

The replicative mechanism of the family *Orthoherpesviridae* requires at least six virus-encoded proteins: a polymerase catalytic subunit and its associated processivity factor, a single-strand DNA binding protein, and a three-protein helicase/primase complex [22]. Structures for the catalytic subunit and processivity factor as well as for the major DNA binding protein have been determined for herpes simplex virus 1 (HSV-1), but no structure has yet been determined for the helicase/primase complex of any herpesvirus [23,24,25,26].

**Figure 2:**
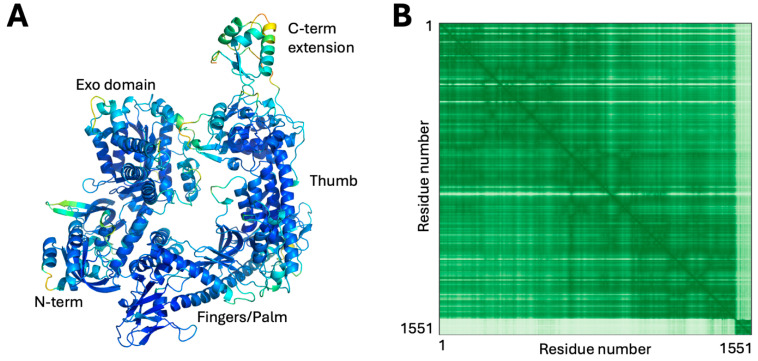
Modeling of ORF58/59. (**A**) Confidence colored model of ORF58/59 with domains indicated. (**B**) PAE plot for the model shown in (**A**). In the scheme for confidence coloring used in this and subsequent figures, blue indicates very high confidence, cyan indicates high confidence, yellow indicates low confidence and orange indicates very low confidence.

SalHV-1 ORF58 was annotated as the DNA polymerase catalytic subunit based on primary sequence similarity, and AlphaFold3 yielded a confident model for ORF58 that gave very high-significance matches to DNA polymerases, including HSV-1 polymerase holoenzyme. The modeled structure lacked a thumb domain, suggesting that the polymerase might be encoded by a spliced gene with the C-terminal portion of the protein in a different exon. Prediction of possible splice donor and acceptor sites within 1 kb of ORF58 using Spliceator v. 2.1 [27] revealed a potential donor site six nucleotides before the ORF58 stop codon and a potential acceptor site 218 nucleotides downstream prior to the annotated start codon of ORF59. This organization is consistent with that observed for other alloherpesviruses, and splicing at these sites would generate a protein that aligns well with the DNA polymerase catalytic subunits from those viruses. Alphafold3 modeling of this sequence yields a highly confident model (Figure 2A,B) with an organization typical for a herpesvirus polymerase, including an N-terminal domain that contains a proofreading exonuclease domain and a fingers-palm-thumb core polymerase. Interestingly, both the HSV and modeled SalHV-1 polymerases have a C-terminal extension beyond the thumb domain (Figure 2A). In the HSV polymerase and others, this extension mediates interaction with a processivity factor [28], suggesting that it may serve a similar function in SalHV-1 ORF58. In the orthoherpesviruses and most dsDNA phages, this processivity factor is encoded by the virus itself (pUL42 in the case of the HSV-1 polymerase). However, no SalHV-1 ORF model yielded a significant match with pUL42 or any other known viral processivity factor. Bacteriophage T7 does not encode its own processivity factor and instead uses the host thioredoxin for this function [29]. SalHV-1 may encode its own novel processivity factor or might hijack a host factor in a similar way to T7.

**Figure 3:**
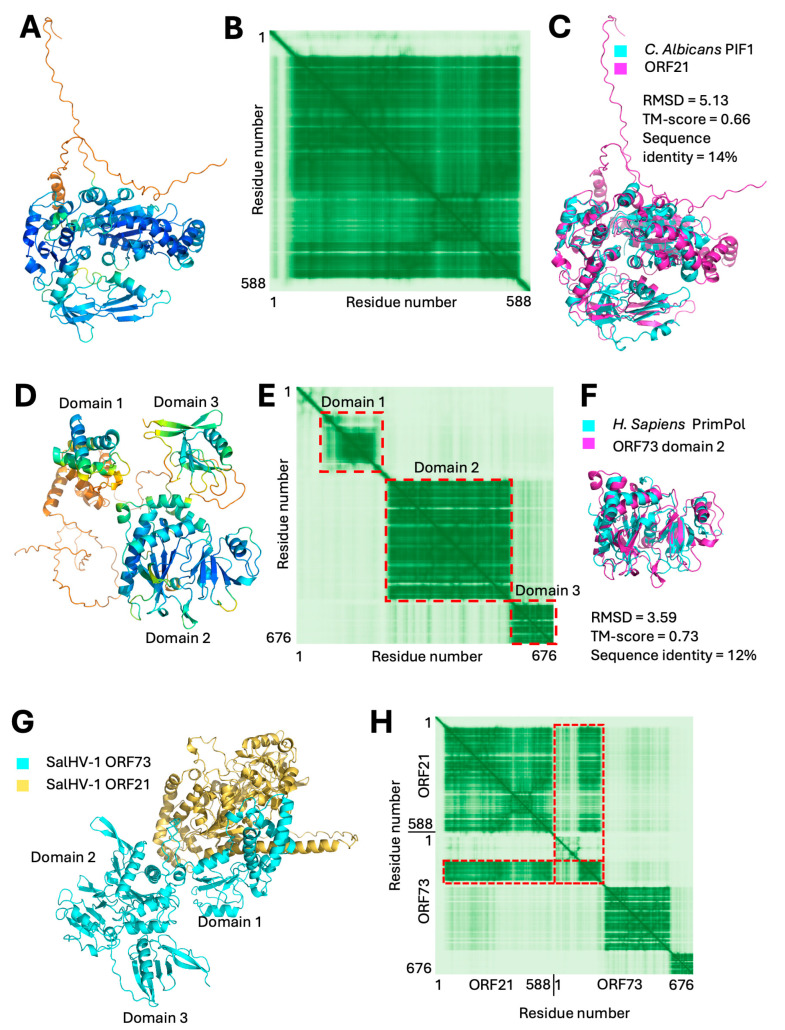
Predicted structures and alignments of helicase/primase complex subunits from SalHV-1. (**A**) Confidence colored AlphaFold3 model of SalHV-1 ORF21. (**B**) PAE plot for the model shown in (**A**). The model consists of a single confidently folded domain flanked by N- and C-terminal disordered extensions. (**C**) Alignment between SalHV-1 ORF21 and *C. albicans* PIF-1 (PDB structure 7OTJ, chain B; DALI Z-score = 26.4, DALI RMSD = 4.3). (**D**) Confidence colored model of ORF73 with putative domains labeled. Domains are separated from each other by disordered regions. (**E**) PAE plot for ORF73 model. Red dashed boxes correspond to domains noted in (**D**). (**F**) Alignment between human PrimPol (PDB 5L2X, chain A) and ORF73 domain 2 (a.a. 248–559). (**G**) Model of complex formed between ORF21 and ORF73 (ipTM = 0.69). Positions of ORF73 predicted domains are indicated. (**H**) PAE plot for the model of the ORF21/ORF73 complex. Red dashed rectangles indicate residues in ORF21 and ORF73 that are confidently placed with respect to each other, and these correspond to the areas of interaction between ORF21 and ORF73 domain 1 shown in (**G**).

The orthoherpesviruses encode their own replicative helicase and primase in a three-subunit heterotrimer (pUL5, pUL8, and pUL52 in HSV) [22,30]. pUL5 is the helicase, pUL52 contains the primase activity, and pUL8 is a scaffold that promotes assembly. A putative helicase (ORF21) could be annotated by sequence similarity, and AlphaFold 3 modeling produced a single-domain structure with high confidence (Figure 3A,B). DALI search found high-significance matches to other helicases, the best match being to the ATP-dependent helicase PIF1 from *Candida albicans* (Figure 3C) [31]. A corresponding primase subunit was found as part of the model of ORF73. The model of this ORF suggested a protein composed of three domains (Figure 3D,E), of which the largest, central domain (a.a. 248–559) showed a very high degree of structural similarity to primases. The best match was with the human PrimPol primase that mediates some aspects of DNA damage repair (Figure 3F) [32,33]. In addition to overall fold similarity shown in Figure 3F, analysis of the overlaid structures revealed conservation of the identity and position of essential active site residues, providing a very strong suggestion that ORF73 is the replicative primase for SalHV-1. Furthermore, each of the three subunits of the helicase/primase heterotrimer of HSV-1 can independently interact with the other two. AlphaFold3 modeled a confident interaction between ORF21 and ORF73 in which most of the interaction between the two subunits is mediated by domain 1 of ORF73 and its N-terminal extension (Figure 3G,H). Iterative PSI-BLAST searches of the viruses taxon using ORF21 and ORF73 as queries returned highly significant matches with ORFs from all other alloherpesviruses with e-values ≤ 1 × 10^−29^ with one (ORF21) or two (ORF73) search iterations and numerous matches with helicase and primase genes from the orthoherpesviruses with two to three iterations. Thus, in addition to the catalytic subunit of the polymerase, the enzymatically active subunits of the helicase/primase complex are shared between the alloherpesviruses and the orthoherpesviruses.

No predicted structure from the SalHV-1 ORFs showed detectable similarity to a model of HSV-1 pUL8. Furthermore, attempts to model trimeric structures with ORF21, ORF73, and each of the other SalHV-1 ORFs failed to produce any model in which the test ORF was confidently placed, suggesting that the helicase/primase complex in the alloherpesviruses may contain only two virus-encoded subunits. In addition, no SalHV-1 ORF models showed similarity to the major single-stranded DNA binding protein of the orthoherpesviruses.

In addition to the essential viral DNA replication machinery, the orthoherpesviruses encode enzymes that promote deoxynucleotide biosynthesis and minimize misincorporation of uracil into the viral genome. These include a ribonucleotide reductase large subunit, a dUTPase, and uracil-DNA-glycosylase [34,35,36,37]. SalHV-1 ORF60 and ORF97 were annotated by sequence similarity as dUTPase and a deoxynucleoside kinase. No proteins with modeled structures similar to ribonucleotide reductase or uracil-DNA-glycosylase were found among the SalHV-1 ORFs.

### 3.3. Capsid Composition and Assembly

The order of *Herpesvirales* and the orders that contain the dsDNA bacteriophages are within the kingdom of *Heunggongvirae*, whose members share a HK97 major capsid protein (MCP) fold. These MCPs form the hexons and pentons of the icosahedral shell. In addition to the MCP, members of the family *Herpesvirales* share with the dsDNA phages a set of capsid assembly and DNA packaging factors. For the capsid, these include a dodecameric portal protein, a scaffold, and maturational protease. For the DNA packaging apparatus, the herpesviruses and the dsDNA phages share a terminase subunit. The orthoherpesviruses share a set of accessory capsid and DNA packaging proteins. Whether these are shared among all members of the family *Herpesvirales* was not clear from primary sequence analysis. In SalHV-1, only three of the capsid proteins, including the MCP (ORF38), the maturational protease (ORF24), and triplex subunit 2 (ORF23) could be annotated based on primary sequence similarity. The portal, scaffold, and triplex subunit 1 were presumed to be present, but the encoding ORFs could not be identified by sequence alone.

**Figure 4:**
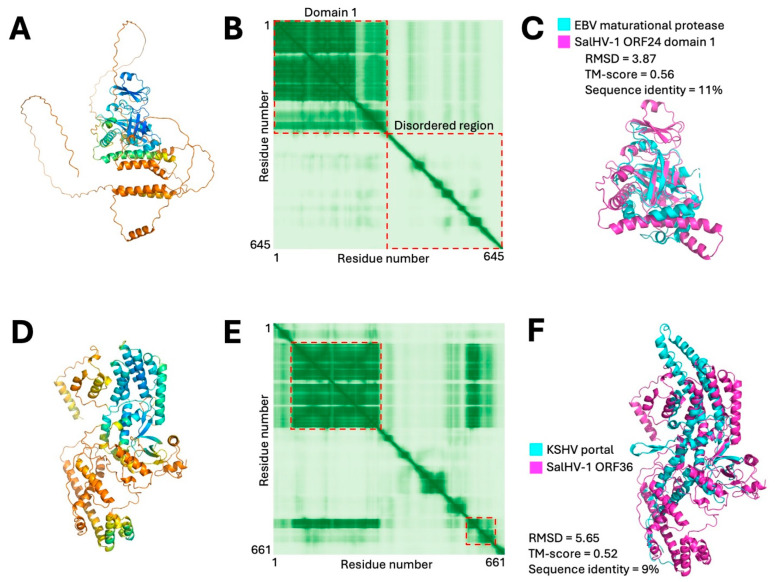
Modeling of SalHV-1 putative scaffold and portal proteins. (**A**) Confidence colored model of ORF24. A confident N-terminal domain is followed by a long, low-confidence region predicted to be largely disordered. (**B**) PAE plot for ORF24 model. Red dashed boxes correspond to the N-terminal ordered domain and the C-terminal disordered region. (**C**) Alignment between EBV maturational protease domain (PDB structure 1O6E, DALI Z-score = 10; RMSD = 3.5) and ORF24 domain 1 (a.a. 1–310). (**D**) Confidence colored model of SalHV-1 ORF36. (**E**) PAE plot for the model shown in (**D**). Red dashed boxes indicate N- and C-proximal regions that come together to form the confidently modeled core region. (**F**) Alignment between KSHV portal monomer subunit (PDB structure 6PPI; DALI Z-score = 9.5; RMSD = 6.6) and the ORF36 model.

HK97 fold capsids are assembled using a scaffold that is cleaved by a maturational protease during genome packaging [38,39]. In the orthoherpesviruses, the maturational protease is part of a larger assemblin protein where the protease comprises an N-terminal ordered domain that is followed by a disordered region that comprises the capsid scaffold. The scaffold region is expressed both as part of the larger protease/scaffold fusion protein, and on its own, from a promoter contained within the sequences that encode the protease catalytic domain [40]. AlphaFold3 modeling of SalHV-1 ORF24 yielded a model with a highly confident, ordered N-terminal domain followed by a 344 residue C-terminal disordered extension (Figure 4A,B). DALI search using the N-terminal ordered domain resulted in three high-significance matches to assembly proteases with the best match being to the EBV protease (Figure 4C). The C-terminal disordered region likely corresponds to the scaffold itself since it is similar in length and domain organization to other orthoherpesvirus scaffolds. Indeed, a PSI_BLAST search found very high-significance matches to ORFs of all other sequenced alloherpesvirus genomes including Anguillid herpesvirus 1 ORF57.5 that was previously annotated as the capsid scaffold based on proteomic analysis of capsids [41].

Another shared feature of all HK97-based capsids is the presence of a dodecameric portal complex through which the genome passes during packaging and serves as a nucleation site for capsid assembly in HSV-1 [42,43,44]. A DALI search of the predicted ORF36 structure identified high-significance matches with both herpesvirus and bacteriophage portal proteins, the best of which was with the KSHV portal protein (Figure 4D–F) [45]. Thus, it is likely that ORF36 is the portal protein homolog in SalHV-1. PSI-BLAST search with ORF36 as the query found highly significant matches (E-values ≤ 1 × 10^−50^) in all sequenced alloherpesviruses.

The orthoherpesvirus capsid includes a heterotrimeric structure called the triplex that sits between MCP hexons and pentons and provides structural support for the very large capsid shell [46]. The two triplex subunits share a similar fold with each other and with homotrimeric capsid cement proteins of some of the dsDNA bacteriophages [47]. ORF23 of SalHV-1 could be annotated as triplex subunit 2 by sequence similarity, and AlphaFold3 produced a confident model with significant matches to triplex 2 subunits of other orthoherpesviruses. Highly confident PSI-BLAST matches to ORF23 could be found in all completely sequenced alloherpesvirus genomes (e values ranged from 2^−69^ to 7^−108^), suggesting that triplex subunit 2 is a core gene of both the orthoherpesviruses and the alloherpesviruses.

**Figure 5:**
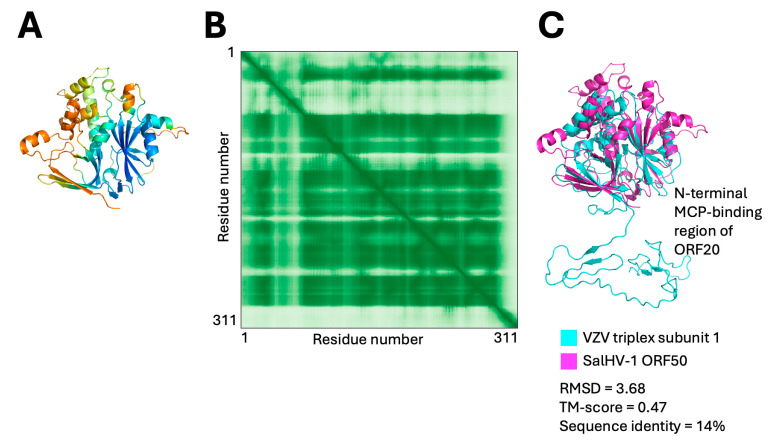
Modeling and alignment of putative capsid triplex proteins. (**A**) Confidence colored model of ORF50. (**B**) PAE plot for the model shown in (**A**). (**C**) Alignment between varicella zoster virus (VZV) triplex subunit 1 and ORF50.

SalHV-1 ORF50 was identified as a likely homolog for triplex subunit 1. The ORF was modeled confidently (Figure 5A,B), and DALI search yielded four high-significance (Z-score > 10) to triplex proteins from VZV, HSV-1, human cytomegalovirus (HCMV), and Epstein–Barr virus (EBV) [48,49,50,51]. Other putative triplex subunits in the alloherpesviruses including ictalurid herpesvirus 2 (ORF53), cyprinid herpesviruses 1 and 2 (ORF66), and anguillid herpesvirus 1 (ORF42) were tentatively annotated based on presence and abundance in the virus capsid [41,52,53]. Indeed, ORF50 shares detectable sequence similarity with the ictaviruses homologs, suggesting that ORF50 encodes SalHV-1 triplex subunit 1. Sequence similarity was not detected between SalHV-1 ORF50 and the putative triplex subunit 1 homologs from Cyprinid HV-1 and -2 and anguillid HV-1, but moderately confident models of these putative homologs from the cyprinid and anguillid herpesviruses could be aligned with SalHV-1 ORF50, suggesting that the triplex subunit 1 is conserved within these genera. A putative homolog could not be identified in the batraviruses.

During HSV capsid assembly, the triplex proteins form a 2:1 heterotrimer that is assembled in the cytoplasm and then transported into the nucleus en bloc and incorporated into the assembling capsid [54,55,56]. Consistent with this behavior, AplhaFold3 will produce highly confident models of the triplex heterotrimers from various members of the family *Orthoherpesviridae*. In contrast, the apparently homologous triplex monomers from SalHV-1, ictaviruses, and cyviruses are not modeled into a heterotrimer with high confidence and the low-confidence models produced bear no resemblance to the triplex models from the orthoherpesviruses. This suggests the possibility that assembly of the alloherpesvirus capsid shell may occur through a different pathway than the one used by the orthoherpesviruses. Interestingly, VZV triplex subunit 1 has a long N-terminal extension that is absent in ORF50 (Figure 5C). This extension interacts extensively with the base of the MCP and is likely critically important for the capsid stabilizing function of the triplex. Its absence in ORF50 suggests that the mode of triplex-MCP interaction in the alloherpesviruses is different.

Finally, the small capsid protein (SCP) decorates the apical tips of MCP hexon subunits, though the occupancy and function vary between the sub-families of the orthoherpesviruses [46]. We could not identify a structural homolog of SCP in any of the predicted SalHV-1 proteins. By itself, this does not strongly suggest that a homologous gene is absent in SalHV-1 or other alloherpesviruses. SCP is the most divergent of the herpesvirus capsid proteins, and sequence or predicted structural homology is not detectable even when comparing across the orthoherpesvirus subfamilies. The SCPs and MCPs of members of the orthoherpesviruses can be confidently modeled as a 1:1 complex, suggesting it might be possible to identify a SalHV-1 SCP by modeling ORF38 with candidate small ORF products. No confident complexes were predicted using this approach with SalHV-1 ORFs less than 250 amino acids, suggesting that either SalHV-1 does not encode an SCP or that it exists but its interaction with the MCP is not confidently predicted. This is consistent with SDS-PAGE analysis of capsid proteins of anguillid herpesvirus 1 and ictalurid herpesvirus 1 which showed no evidence for a small capsid protein [41,52].

### 3.4. DNA Packaging

In most double-strand DNA phages, the terminase is composed of a pentamer of heterodimers where one subunit recognizes the viral genome and the second subunit cleaves the genome and translocates it through the portal [57]. In contrast, the orthoherpesviruses encode a tripartite terminase. One subunit (subunit 1; pUL15 in HSV) contains an N-terminal RecA-like ATPase domain and C-terminal RNase H-like endonuclease domain homologous to bacteriophage terminases. The other two subunits, one large (subunit 2; pUL28 in HSV) and one small (subunit 3; pUL33 in HSV), are specific to herpesviruses.

**Figure 6:**
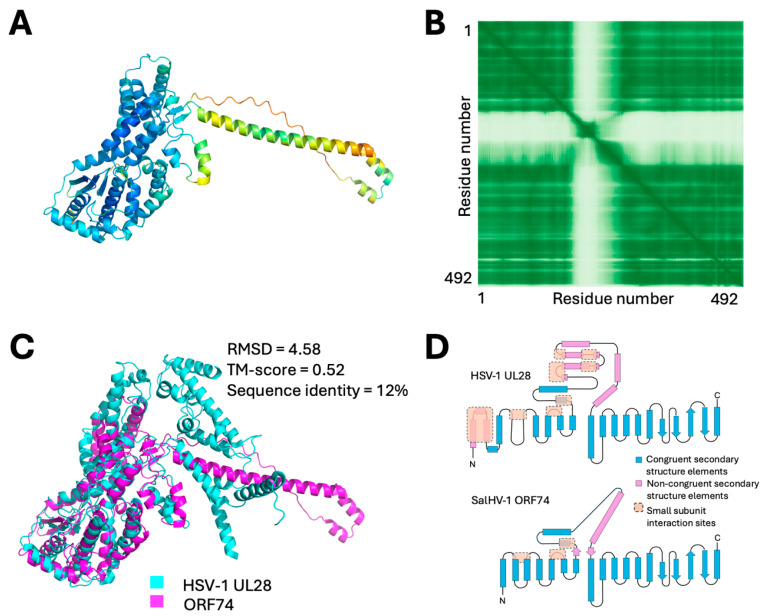
Modeled structures and alignments of putative SalHV-1 terminase subunit 2. (**A**) Confidence colored model of SalHV-1 ORF74. (**B**) PAE plot for the model shown in (**A**). (**C**) Alignment of HSV-1 UL28 (PDB structure 6M5R, chain B; DALI Z-score 21.5, RMSD 3.7) with SalHV-1 ORF74. (**D**) Fold topology diagrams for HSV pUL28 and SalHV-1 ORF74.

A homolog to subunit 1 of the terminase was found by primary sequence similarity. The coding sequence is split between ORFs 72, 80, and 82 that correspond to its three exons. These sequences produced a confident model that aligned well with the HSV-1 subunit 1 structure. While a homolog to terminase subunit 2 in SalHV-1 was not evident in sequence similarity searches, we identified ORF74 as a candidate. ORF74, a 492 a.a. protein, has N- and C-terminal regions that are modeled into a single high-confidence domain, while the central region (a.a. 166–268) is modeled as a low-confidence alpha-helical protrusion (Figure 6A,B). Structural homology searches with ORF74 produced a unique and highly significant match to subunit 2 of the HSV-1 tripartite terminase (Figure 6C). The central regions of ORF74 and pUL28 align very poorly, but the N-/C-domain regions align very well (Figure 6C). The central regions also differ considerably in length, accounting for most of the difference in overall length of the proteins. PSI-BLAST search with ORF74 revealed highly significant matches (e-values ≤ 9 × 10^−81^) in all sequenced alloherpesviruses, suggesting that this is a core gene in both allo- and orthoherpesviruses.

No homolog to terminase subunit 3 was detected among the SalHV-1 ORFs. This subunit exclusively interacts with subunit 2 in the terminase of HSV [58]. It is possible that SalHV-1 encodes a homolog of subunit 3 that was not detected because it is too small to be confidently modeled, or it does not adopt a unique conformation outside of its complex with ORF74, or both. However, two observations suggest that SalHV-1 may truly lack a subunit 3 homolog. First, despite their short length, AlphaFold 3 returns very confident models of the small subunits of the homologs in HSV, HCMV, and EBV that are very similar to the experimentally observed structure in the HSV tripartite terminase. Second, as noted above, the terminase subunit 2 of the orthoherpesviruses has a long central region that is absent in ORF74 of SalHV-1 (Figure 6C,D). Most of the structural elements in HSV-1 subunit 2 that mediate interaction with subunit 3 are missing in SalHV-1 ORF74, suggesting that the subunit 3 homolog is missing as well.

**Figure 7:**
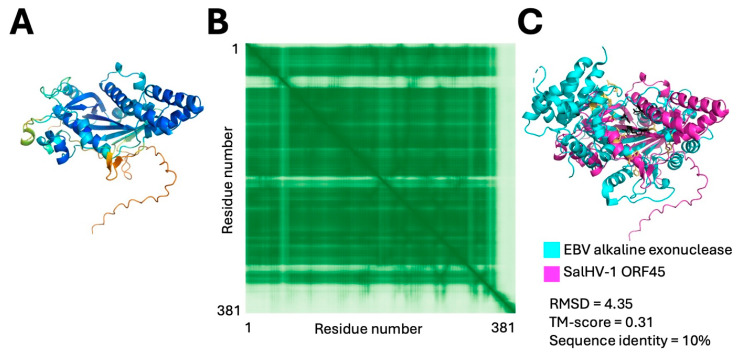
Modeled structure and alignment of ORF45 putative genome packaging exonuclease. (**A**) Confidence colored model of ORF45. (**B**) PAE plot for ORF45 model. (**C**) Alignment between the EBV alkaline exonuclease (RCSB PDB 2W4B) and the ORF45 model.

In addition to the terminase, members of the family *Orthoherpesviridae* encode two accessory proteins that are necessary for efficient genome packaging. One is a pentameric packaging factor (pUL32 in HSV-1) of unclear function. No protein with a predicted structure similar to pUL32 was detected in the predicted SalHV-1 ORFs. The other accessory protein is an exonuclease (pUL12 in HSV-1) that helps process the viral genomic DNA. The exonuclease is not absolutely required for HSV replication, but a null mutant or mutant that lacks catalytic activity is severely impaired for replication, produces fewer DNA-containing capsids, and the DNA-containing capsids produced contain aberrant genomes [59,60,61,62,63,64]. SalHV-1 ORF45 was modeled very confidently (Figure 7A,B), and the protein model yielded high Z-score (>8.0) matches with alkaline exonuclease structures, including several dsDNA bacteriophage alkaline exonucleases and, notably, the alkaline exonuclease from EBV (Figure 7C). Nucleases of this family share a conserved catalytic D-(D/E)-X-K motif that is present in ORF45 suggesting that it encodes a functional nuclease. The ORF45 sequence is conserved among the sequenced alloherpesviruses (E-values for PSI-BLAST search ≤ 5 × 10^−35^). The presence of this homolog in the alloherpesviruses suggests that members of the order *Herpesvirales* may all use a similar genome processing strategy derived from the common ancestor of DNA phages and herpesviruses.

**Figure 8:**
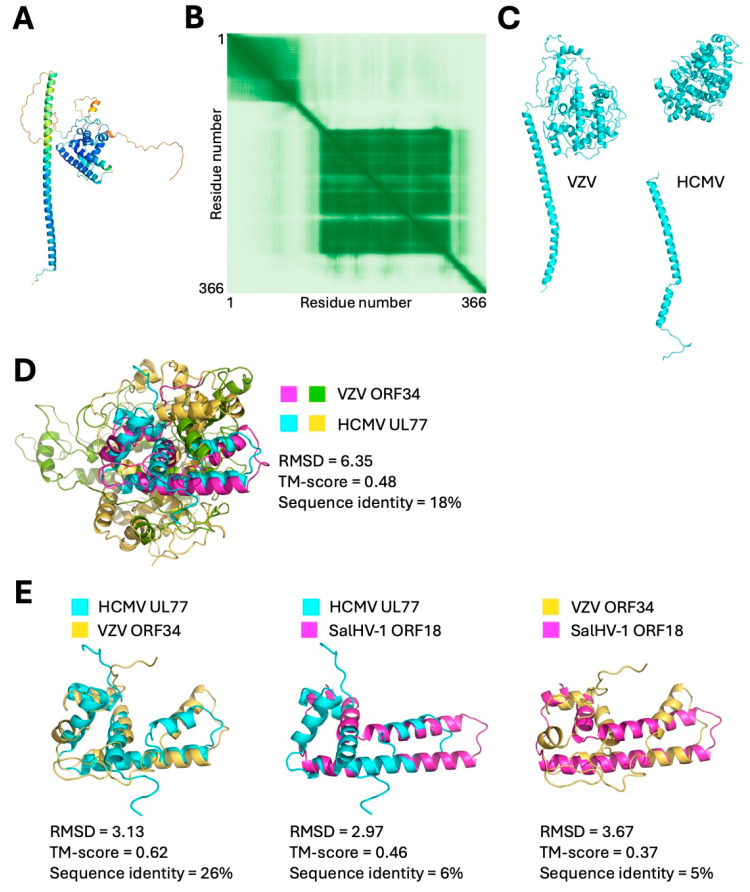
Modeled structure and alignments of SalHV-1 ORF18 with putative HCMV and VZV homologs. (**A**) Confidence colored model of ORF18. (**B**) PAE plot for ORF18 model. (**C**) Experimentally determined structures for VZV and HCMV CSVC component 2 from PDB 9NO1 and 8TEU, respectively. (**D**) Alignment between the globular domains of VZV ORF34 (magenta and green) and HCMV UL77 (cyan and gold). (**E**) Isolated alignments of the C-terminal regions of close alignment between HCMV UL77 (cyan), VZV ORF34 (gold), and SalHV-1 ORF18 (magenta).

Completion of DNA packaging is correlated with association of a capsid vertex-specific complex (CVSC) with the penton vertices. The CVSC is composed of two proteins (called pUL17 and pUL25 in HSV-1) that provide an anchoring point for the proteins of the inner tegument, beginning with the large tegument protein [45,47,65,66]. Determination of the structure of the HCMV portal vertex from mature virions reveals that the pUL25 homolog (pUL77) “caps” the portal and its association with the vertex is reinforced by its association with the pUL17 homolog (pUL93), which binds both to pUL77 and to anchor points on the MCP subunits of the penton [65]. Structural prediction and search revealed a possible homolog for only one of the two proteins of the CVSC in SalHV-1. ORF18 of SalHV-1 is predicted to form a long alpha helix followed by a confidently modeled globular domain (Figure 8A,B). DALI search with the confidently modeled portion of the protein yielded a relatively low-significance match to HCMV pUL77, which has the same overall organization (Figure 8C) [65]. Among the orthoherpesvirus homologs, only UL77 and its VZV homolog, ORF34, have experimentally determined structures that include the globular domain [65,67]. Interestingly, structural alignment between the globular domains of HCMV UL77 and VZV ORF34 is relatively poor except for the most C-terminal portion (a.a. 521–641 of UL77 and a.a. 459–576 of ORF34) (Figure 8D,E). Close alignment of the SalHV-1 ORF18 model to the VZV and HCMV structures is confined to the same region (Figure 8E). It seems likely, therefore, that ORF18 is homologous to UL77, ORF34, and their homologs in the orthoherpesviruses and may serve a similar portal cap function. PSI-BLAST search using the ORF18 sequence as query also yields highly significant matches (E-value ≤ 4 × 10^−32^) among all the sequenced alloherpesviruses, suggesting a critical conserved core function.

### 3.5. Nuclear Egress

Nuclear egress in the orthoherpesviruses is mediated by a heterodimeric complex of two proteins (pUL31 and pUL34 in HSV) that recruit kinases for disruption of the nuclear lamina, bind the nucleocapsid, and accomplish membrane curvature by formation of hexameric arrays [68,69]. pUL31 is a nucleoplasmic protein that is recruited to the inner nuclear membrane by interaction with pUL34, a type II integral membrane protein. In addition to their interactions that result in heterodimerization, formation of hexamers is mediated mostly by interactions between the pUL31 and pUL34 subunits of adjacent heterodimers [70,71]. These two proteins are thus completely dependent upon each other for function in nuclear egress.

**Figure 9:**
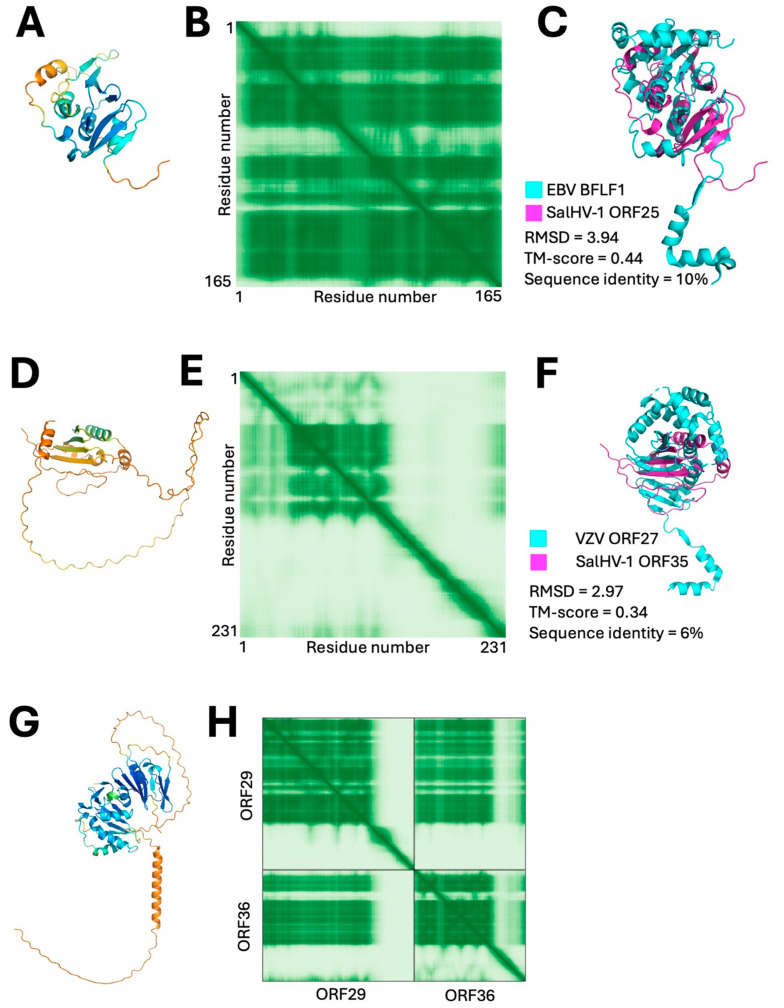
Putative nuclear egress complex protein homologs in SalHV-1. (**A**) Confidence colored model of putative NEC subunit 1 homolog, ORF25. (**B**) PAE plot for ORF25 model. (**C**) Alignment between SalHV-1 ORF25 and EBV BFLF2 (RCSB PDB 7T7I chain D; DALI Z score of 6.9 and RMSD = 5.0). (**D**) Confidence colored model of putative pUL34 homolog, ORF35. (**E**) PAE plot for ORF35 model. (**F**) Alignment between SalHV-1 ORF35 and VZV ORF27 (RCSB PDB 7PAB chain A). (**G**) Confidence colored model of putative complex between Ictalurid herpesvirus 2 proteins ORF29 (SalHV-1 ORF25 homolog) and ORF36 (SalHV-1 ORF35 homolog). (**H**) PAE plot for complex model shown in (**G**) indicating high confidence placement of globular domains with respect to each other.

Two SalHV-1 ORFs (ORF25 and ORF35) showed structural similarity to the pUL31 subunit of the NEC. ORF25 was modeled with moderate confidence overall and a very confidently modeled central core (Figure 9A,B). DALI identified structural homology to orthoherpesvirus UL31 homologs, with the best match to the EBV homolog BFLF2 (Figure 9C) [72]. The RCSB pairwise alignment shows good alignment of the core beta sheets and some of the surrounding alpha helices. The ORF25 model, however, lacks some of the alpha-helical structures conserved in UL31 homologs, including C-terminal helices that make up the most membrane distal part of the NEC and the N-terminal hook that provides most of the binding surface for the heterodimerization with UL34. BLAST search for ORF25 homologs among the other alloherpesviruses yielded significant matches only with proteins from ictaviruses. Additional homologs in the batraviruses were found using a positional homology strategy. SalHV-1 ORF25 is adjacent to ORF24, which encodes the putative assembly protease/scaffold protein and whose homologs can be found throughout the alloherpesviruses. AlphaFold 3 modeling of ORF24 homolog-adjacent ORFs revealed high-significance structural matches in Ranid herpesviruses1, 2, and 3, ORFs 62, ORF87, and 81, respectively. Homologs were not found using this strategy in the cyviruses, possibly because positional homology is not conserved in this subfamily.

While we were unable to identify an obvious homolog for UL34, we identified a second UL31-like gene. SalHV-1 ORF35 was modeled with relatively low confidence (Figure 9D,E), but, interestingly, the model predicted an N-terminal ordered domain followed by a C-terminal disordered domain that terminates in a predicted helix, enriched in hydrophobic amino acids that might form a membrane anchoring helix. This overall organization is like that observed for the UL34 subunit of the NEC heterodimer. DALI search using the N-terminal ordered domain as the query did not yield any matches with UL34 homologs but did identify the same three UL31 homologs with structural homology to ORF25, albeit with substantially lower significance. None of the three UL31 homologs were in the top ten matches, and Z-scores were all below six. It has been previously noted that pUL31 and pUL34 share the same basic “beta taco” core fold: two anti-parallel beta-sheets face each other at an angle to form the shell with a filling composed of side chains that extend inward from the sides of the shell and a “topping” layer composed of alpha helices [70]. This similarity suggested that pUL31 and pUL34 might be paralogous proteins that arose from a gene duplication followed by structural and sequence divergence. The similarity in domain organization between UL34 and SalHV-1 ORF35 along with the weak but identifiable structural similarity between ORF35 and UL31 suggest the possibility that ORF35 might be homologous to both pUL31 and pUL34 homologs and function as the integral membrane subunit of the NEC. However, the alignment between ORF35 and VZV ORF27, its closest match among UL31 homologs, only corresponds to half of the “taco” shell with connected “topping” alpha helices (Figure 9F). BLAST search for homologs of ORF35 yielded no significant hits, but positional homology to the adjacent putative portal protein (ORF36) allowed us to identify homologs in all ictaviruses and a batravirus (Ranid herpesvirus 1). Interestingly, the putative homologs of ORF35 in the ictaviruses include both halves of the “taco shell,” suggesting that ORF35 of SalHV-1 may correspond to a 3′ exon rather than the complete protein coding sequence. We were, however, unable to detect a more 5′ exon by spice site prediction or search for short ORFs with sequence similarity to the N-terminus of ictavirus homologs. SalHV-1 ORF25 and 35 cannot be modeled confidently as a complex, perhaps because an N-terminal part of ORF35 is missing. However, the homologous proteins from ictalurid herpesvirus 2 (ORF29 for ORF25 and ORF36 for ORF35) which include a complete taco shell for the ORF36 homolog, can be modeled as a heterodimer with high confidence (Figure 9G,H). Taken together, these observations suggest that at least SalHV-1 and the ictaviruses encode homologs for the heterodimeric NEC.

In addition to the pUL31/pUL34 nuclear egress complex, other proteins conserved among the orthoherpesviruses have been shown to play important roles in nuclear egress through regulation of phosphorylation at the nuclear envelope. These include pUL21 (in HSV-1 and HSV-2) and pUL16 (in HSV-2), which form a complex with each other in the alphaherpesviruses [73,74,75] and the conserved herpesvirus protein kinase (CHPK) (pUL13 in HSV-1 and 2, BGLF4 in EBV, and pUL97 in HCMV) [76,77,78,79,80]. No AlphaFold models of SalHV-1 ORFs gave significant matches to either pUL16 or pUL21. DALI search using SalHV-1 models suggested that five different ORFs are likely to encode protein kinases or related proteins (ORFs 20, 83, 85, 119, and 120). Which, if any, of these corresponds to the orthoherpesvirus CHPK could not be determined since only the putative catalytic domains exhibited detectable similarity.

### 3.6. Cytoplasmic Assembly and Entry

Upon egress of capsids from the nucleus, the capsid acquires inner tegument proteins in the cytosol and the partially tegumented capsid is then enveloped by cytoplasmic membranes that contain the envelope glycoproteins and associated outer tegument proteins [81,82]. Members of the family *Orthoherpesviridae* encode many tegument and envelope proteins (>30 in HSV-1). None of the AlphaFold models of SalHV-1 ORFs gave significant matches to any of these proteins suggesting that the SalHV-1 tegument and envelope may have an entirely different composition and, further, that its mechanism of cytoplasmic assembly is based on a different set of interactions.

## 4. Discussion

**Core orthoherpesvirus genes in SalHV-1 and the alloherpesviruses.** Structural modeling and structural similarity searches described here confirmed and extended the list of herpesvirus core genes found in the family *Alloherpesviridae* (Table 1). The basic DNA replication apparatus including catalytic polymerase and helicase is extended to include a primase subunit, but not a helicase primase accessory subunit, a polymerase processivity factor, or a single-strand DNA binding protein. Homologs of all capsid proteins (except for the small capsid protein) were detected, but with interesting predicted structural differences in the triplex subunits that suggest a modified capsid assembly mechanism. The similarities in DNA packaging were extended to include the intermediate subunit of the terminase, the alkaline exonuclease, and the capsid vertex specific component. Homologs of these proteins could be identified in all other sequenced alloherpesvirus genomes (except for capsid triplex 1 that could not be identified in the batraviruses). Putative homologs to both subunits of the NEC could be found, although homologs were not identifiable by sequence similarity in some other alloherpesviruses.

Many genes that are conserved among the *Orthoherpesviridae* could not be identified either by sequence or structural homology searches, including some components of the replication apparatus, genome packaging machinery, and all the tegument and envelope proteins. Some of these “absent genes” represent orthoherpesvirus-specific elaborations of mechanisms whose basic elements are shared with the dsDNA bacteriophages (e.g., the HSV-1 UL8 accessory subunit of the replicative helicase/primase, the HSV-1 UL33 small subunit of the DNA packaging terminase, and the HSV-1 UL32 packaging protein). Interestingly, the functions and fitness advantages provided by these accessories in the orthoherpesviruses are not clear. While it is possible that these elaborations were present in the common ancestor of the *Herpesvirales* and were subsequently lost during evolution of the alloherpesviruses, a simpler explanation is that they were acquired over time, specifically by the orthoherpesviruses.

The most surprising element of our analysis was the failure to detect any homologs of the tegument or envelope proteins of the orthoherpesviruses. The virions of the alloherpesviruses are morphologically typical, and assembly intermediates like those seen during replication of orthoherpesviruses are observed in alloherpesvirus infection [6,7]. Nonetheless, the molecular interactions that mediate those assembly events must be very different and represent an alternative approach to construction of these very complex particles. The absence of homologs to orthoherpesvirus envelope proteins, especially the three-protein gB-gH/gL core entry apparatus, also suggests a quite different entry strategy. The fusion protein of the orthoherpesviruses (gB) is a class III fusion protein [8]. It was previously observed that a putative membrane glycoprotein of Anguillid herpesvirus 1 virus shows significant sequence similarity to class I fusion proteins like the coronavirus spike, suggesting that the alloherpesviruses may use an entirely different and much simpler fusion apparatus [41].

It should be noted that the structural similarity search approach can fail to identify homologous genes for any of several reasons, including the following: (i) the gene encodes a protein that is intrinsically disordered; (ii) the current annotation is missing exons that encode critical structural elements; (iii) the protein lacks sufficient structural complexity to generate a significant match (for example, a protein composed of a single long alpha helix). For example, we failed to detect a homolog for the HSV-1 tegument protein pUL11, which is conserved among the orthoherpesviruses, but that is intrinsically disordered [83]. However, these factors are unlikely to account for failure to detect many orthoherpesvirus core genes in SalHV-1. The major single-stranded DNA binding protein, polymerase processivity factor, pentameric genome packaging accessory factor, and most of the conserved tegument proteins, for example, are relatively large, structurally complex proteins that would need to be split among many exons to evade detection. It seems likely, therefore, that they are truly missing in SalHV-1 and perhaps in the other alloherpesviruses.

It is tempting to suggest that the absence in the alloherpesviruses of some genes that are conserved in the orthoherpesviruses indicates a simpler replication mechanism that is closer to that of the common ancestor of the order *Herpesvirales.* However, our analysis of the SalHV-1 genome did not identify homologs or suggest obvious functions for many of the annotated ORFs. It is entirely possible that these encode different, but equally elaborate accessories for basic replication mechanisms. In either case, detailed study of genome replication and DNA packaging processes in the alloherpesviruses might provide interesting and significant insights into the adaptations that accompanied the transition of large DNA viruses from bacterial to eukaryotic hosts.

## Figures and Tables

**Table 1:** Conserved genes of the orthoherpesviruses and homologs in SalHV-1 and alloherpesviruses ^a^.

Mechanism Group	Protein Structure Group/Function (HSV Gene)	SalHV-1	Other Allo ^b^
Envelope glycoproteins	Envelope glycoprotein B (UL27) Envelope glycoprotein LH (UL22) Envelope glycoprotein L (UL1) Envelope glycoprotein M (UL10) Envelope glycoprotein N (UL49)	ND ^c^ ND ND ND ND	NR ^d^ NR NR NR NR
Regulatory	DNA-binding/transcription (UL3) Post-transcriptional regulation (ICP27) Protein kinase (UL13)	ND ND ND	NR NR NR
Genome replication/integrity	DNA polymerase catalytic subunit (UL30) Major single-stranded DNA-binding protein (UL29) Polymerase processivity factor (UL42) Helicase/primase helicase (UL5) Helicase/primase primase (UL52) Primase-associated factor (UL8) Uracil-DNA glycosylase (UL2) dUTPase (UL50) Ribonucleotide reductase large subunit (UL39)	ORF58/59 ND ND ORF21 ORF73 ND ND ORF60 ND	All NR NR All All NR Cyviruses All Cyviruses
Capsid structure/assembly	Major capsid protein (UL19) Triplex protein 1 (UL38) Triplex protein 2 (UL18) Small capsomere interacting protein (UL35) Portal protein (UL6) Maturational protease/scaffold (UL26/26.5)	ORF38 ORF50 ORF23 ND ORF36 ORF24	All All except batraviruses All NR All All
DNA packaging	Terminase subunit 1 (UL15) Terminase subunit 2 (UL28) Tripartite terminase subunit 3 (UL33) packaging protein (UL32) Alkaline exonuclease (UL12) Capsid vertex component 2 (UL25)	ORF72/80/82 ORF74 ND ND ORF45 ORF18	All All NR NR All All
Nuclear egress	Nuclear egress 1 (UL31) Nuclear egress 2 (UL34)	ORF25 ORF35	Ictaviruses; batraviruses Ictaviruses; Ranid HV-1
Tegument	Capsid vertex component 1 (UL17) Large tegument protein deneddylase (UL36) Inner tegument protein (UL37) Tegument protein (UL21) Cytoplasmic envelopment protein (UL16) Cytoplasmic envelopment protein (UL11) Cytoplasmic envelopment protein (UL7) Cytoplasmic envelopment protein (UL51)	ND ND ND ND ND ND ND ND	NR NR NR NR NR NR NR NR

^a^ Protein structure group/function designations are the same as in Herpesfolds [13]. ^b^ Detected by sequence homology search in full genome sequences of isolated alloherpesviruses in this or other studies. ^c^ Not detected in this study. ^d^ Not reported in the literature.

## Data Availability

The SalHV-1 complete genome is available on GenBank (OK337613) together with sequencing raw data (SRR16095036-SRR16095036), under BioProject PRJNA766810.

## Abbreviations

The following abbreviations are used in this manuscript:

CVSC	Capsid vertex-specific complex
EBV	Epstein–Barr virus
HCMV	Human cytomegalovirus
HSV	Herpes simplex virus
IR	Internal repeat
IRS	Internal repeat sequence
MCP	Major capsid protein
NEC	Nuclear egress complex
ORF	Open reading frame
PAE	Predicted aligned error
PDB	Protein Data Bank
RCSB	Research Collaborative for Structural Bioinformatics
SalHV-1	Salmonid herpesvirus 1
SCP	Small capsid protein
TRS	Terminal repeat sequence
UL	Unique long
US	Unique short
VZV	Varicella zoster virus
